# The Relationship between LRP6 and Wnt/β-Catenin Pathway in Colorectal and Esophageal Cancer

**DOI:** 10.3390/life13030615

**Published:** 2023-02-23

**Authors:** Akemi Shishido, Masaaki Miyo, Kazuki Oishi, Natsumi Nishiyama, Meiqiao Wu, Hiroyuki Yamamoto, Shihori Kouda, Xin Wu, Satoshi Shibata, Yuhki Yokoyama, Hirofumi Yamamoto

**Affiliations:** 1 Department of Molecular Pathology, Division of Health Sciences, Graduate School of Medicine, Osaka University, Yamadaoka 1-7, Suita City 565-0871, Japan; 2 Department of Surgery, Gastroenterological Surgery, Graduate School of Medicine, Osaka University, Yamadaoka 2-2, Suita City 565-0871, Japan

**Keywords:** LRP6, colorectal cancer, Wnt signaling, esophageal squamous cell carcinoma

## Abstract

High expression of low-density lipoprotein receptor-related protein 6 (LRP6), a key component of the Wnt/β-catenin signaling pathway, is reported to be associated with malignant potential in some solid tumors including breast cancer and hepatocellular carcinoma. Few reports, however, have examined its function and clinical significance in colorectal cancers (CRC) demonstrating constitutive activation of Wnt signaling. Here, we compared the expression level and function of LRP6 in CRC with that of esophageal squamous cell carcinoma (ESCC) bearing few Wnt/β-catenin pathway mutations. On immunohistochemical staining, high LRP6 expression was noted in three of 68 cases (4.4%), and high β-catenin in 38 of 67 cases (56.7%) of CRC. High LRP6 expression was found in 21 of 82 cases (25.6%), and high β-catenin expression in 29 of 73 cases (39.7%) of ESCC. In our in vitro studies, LRP6 knockdown hardly changed Wnt signaling activity in CRC cell lines with mutations in Wnt signaling downstream genes. In contrast, in ESCC cell lines without Wnt signaling-related mutations, LRP6 knockdown significantly decreased Wnt signaling activity. LRP6 function may depend on constitutive activation of Wnt signaling.

## 1. Introduction

In epithelial tissue, the processes of cell proliferation and differentiation are coordinately regulated [1,2]. One of the most pivotal signaling pathways associated with this regulation is the Wingless and INT-1 (Wnt)/β-catenin pathway [3]. β-catenin is a subunit of the cadherin protein complex, whose stabilization is crucial for the activation of Wnt/β-catenin signaling [4]. In the absence of Wnt, β-catenin is phosphorylated by a protein complex consisting of adenomatous polyposis coli (APC), AXIN, glycogen synthase kinase-3β, and casein kinase 1, followed by ubiquitin-mediated degradation of β-catenin in the proteasome [5]. This active degradation mechanism keeps cytoplasmic β-catenin concentrations consistently low [6].

In the presence of Wnt, Wnt ligands bind to the seven-pass transmembrane receptor Frizzled and the low-density lipoprotein receptor-related protein (LRP) 6, inducing LRP6 phosphorylation. The active form of LRP6 then binds by phosphorylation to AXIN, suppressing degradation of β-catenin and releasing it into the cytoplasm [7]. β-catenin accumulates in the cytoplasm and translocates into the nucleus and binds to the TCF/LEF (T-cell factor/lymphoid enhancer factor) transcription factor to promote expression of genes that regulate cell cycle, proliferation, survival, and differentiation [8] (Supplementary Figure S1).

The abnormal activation of Wnt/β-catenin pathway by gene mutation is involved in the pathogenesis of various diseases, especially human cancers [3]. For example, a high incidence of mutations in the β-catenin gene (*CTNNB1*) has been observed in liver cancer [9], endometrial cancer [10], and colorectal cancer (CRC) [11]. Mutations in *APC*, a key tumor suppressor gene, have been detected in most CRCs [12], as well as in some other cancers including gastric cancer [13].

LRP6 is an indispensable transmembrane receptor for Wnt and is overexpressed in several solid tumors, including colorectal [14], liver [15], and breast cancers [16], as well as pancreatic adenocarcinomas [17], in association with increased Wnt/β-catenin signaling.

Increased LRP6 phosphorylation involved in nuclear accumulation of β-catenin also has been observed in CRC, with relevance for tumor malignancy and staging [18], along with poor prognosis [19]. In addition, in a study using whole-exome sequencing, functional variants of LRP6 were identified as novel risk factors for early-onset CRC [20]. With increasing evidence that LRP6 is closely associated with tumor development and progression, various antibodies [21], peptides [16], and small molecules [22] have been developed and show anticancer properties by inhibiting LRP6 function directly or indirectly.

Although the aberration of LRP6 is observed in many types of cancers with various gene mutation frequencies in the Wnt/β-catenin pathway, there are no reports showing whether LRP6 could behave differently in cancers based on Wnt/β-catenin pathway genetic abnormalities. For this reason, the purpose of this study was to elucidate the role of LRP6 in CRC and esophageal squamous carcinoma (ESCC), which shows a low frequency of gene mutations in the Wnt/β-catenin pathway [23], and no reports address LRP6 function.

## 2. Materials and Methods

### 2.1. Cell Lines and Culture

Human CRC cell lines (DLD-1, with truncated *APC* and wild-type β-catenin; HCT116, with a Ser45 deletion in one β-catenin allele and one wild-type allele, wild-type *APC*) and ESCC cell lines (TE-1 and TE-8: wild-type *APC*; wild-type β-catenin) were purchased from the American Type Culture Collection (ATCC, Manassas, VA, USA). These cell lines were authenticated by morphologic inspection, short tandem repeat profiling, and mycoplasma testing by the ATCC. The authors also performed the mycoplasma testing on a regular basis. Cell lines were maintained in Dulbecco’s modified Eagle’s medium and Roswell Park Memorial Institute 1640 with 10% fetal bovine serum and 100 U/mL penicillin and 100 μg/mL streptomycin in humid 5% CO_2_ at 37 °C.

### 2.2. Clinical Tissue Samples

CRC samples were collected from 68 patients and ESCC samples from 82 patients who underwent surgery at Osaka University Hospital (Osaka, Japan) between 2006 and 2010. As positive controls, we used samples from three cases of breast cancer. Written informed consent was obtained from all patients, in accordance with guidelines approved by the Institutional Research Board. This study was conducted under the supervision of the Ethics Board of Osaka University Hospital (No. 15144).

### 2.3. siRNA Transfection

Small interfering (si)RNA against LRP6 (siRNA-LRP6) and negative control siRNA (NC-siRNA) were purchased from GeneDesign Inc. (Osaka, Japan). Transfection was performed at a final concentration of 30 nM with Lipofectamine 2000 (Thermo Fisher Scientific, Waltham, MA, USA), based on the manufacturer’s protocol.

### 2.4. Immunohistochemical Staining

Formalin-fixed, paraffin-embedded tissues were prepared and sectioned into 4 μm slices. Sections were deparaffinized with xylene and then rehydrated in graded alcohols. Immunostaining was performed using antibodies and the VECTASTAIN Elite ABC Kit (Vector Laboratories, Burlingame, CA, USA), according to the manufacturer’s protocol. Anti-LRP6 rabbit polyclonal antibody (PA5-13145) was obtained from Thermo Fisher Scientific, and anti-β-catenin mouse monoclonal antibody (610153) was obtained from BD Transduction Laboratories (Franklin Lakes, NJ, USA). The stain intensity was graded as 0 to 2 (0 = none; 1 = weak; 2 = strong), and the percentage of positive cells was scored as 1 to 3 (1 = 0–49%; 2 = 50–89%; 3 = 90–100%). The two scores were multiplied together to determine a staining score, with possible scores of 0, 1, 2, 3, 4, or 6. Immunostaining for the LRP6 protein was performed in 68 sets of normal colon tissues and cancer tissues and 82 sets of normal esophageal tissues and cancer tissues. The breast cancer tissue served as a positive control for the primary antibody (Supplementary Figure S2A). A serial section in which phosphate-buffered saline was used instead of the primary antibody served as a negative control (Supplementary Figure S2B). Immunostaining for the β-catenin protein was performed using 67 CRC samples and 73 ESCC samples. The 58 and 68 samples of CRC and ESCC were used for the clinicopathological analysis and survival analysis, respectively. For the relapse-free survival analysis, stage IV cases were omitted.

### 2.5. Western Blot Analysis

The protein samples were subjected to SDS-PAGE and transferred onto polyvinylidene difluoride membranes. Cell lysates and the antibodies against LRP6 (C47E20) and β-actin (ACTB) were obtained from Cell Signaling Technology, and the antibody against β-catenin (610153) from BD Transduction Laboratories. We collected protein lysate after processing by siRNA at 48 and 72 h. After incubation with secondary antibodies, signals were detected with the Pierce Western Blotting Substrate (Thermo Fisher Scientific).

### 2.6. RNA Isolation and qRT-PCR

Total RNA from cell lines was isolated using TRIzol reagent (Thermo Fisher Scientific) following the manufacturer’s protocol. RNA concentration and purity were assessed with a Nano Drop One spectrophotometer W1F1 (Thermo Fisher Scientific). Total RNA was reverse transcribed using the High-Capacity RNA-to-cDNA kit (Thermo Fisher Scientific). To measure LRP6 expression level, a real-time quantitative polymerase chain reaction (qRT-PCR) was performed using a LightCycler 2.0 Instrument (F Hoffmann-La Roche AG, Basel, Switzerland) with LightCycler TaqMan Master (F Hoffman-La Roche AG). The product numbers of the TaqMan Gene Expression assay were as follows: ACTB ID Hs01060665_g1 and LRP6 ID Hs00999795_m1. Relative expression was quantified using the ΔΔct method.

### 2.7. TOPFLASH Assay

Cells were seeded onto 96-well plates (DLD-1, TE-8: 4000 cells/well; HCT116, TE-1: 3000 cells/well), and transfection of siRNA was performed using Lipofectamine 2000 (Thermo Fisher Scientific). At 24 h after siRNA transfection, cells were transfected with 100 ng/well of the TOPFLASH luciferase reporter vector (pGL4.49 [luc2P/TCF-LEF RE/Hygro]; Promega, Fitchburg, WI, USA) using Lipofectamine 2000 and treated with 100 ng/mL human recombinant Wnt3a (R&D Systems, Minneapolis, MN, USA). After 24 h, cells were collected with the reporter lysis buffer for luciferase activity measurement using the Dual-Luciferase Reporter Assay System (Promega). Firefly luciferase activity was normalized against Renilla luciferase activity for each transfected well. The transfection efficiency was evaluated with 1 ng/well of the Renilla luciferase reporter vector (pRL-CMV, Promega). We incubated four kinds of cultured cells under unstimulated conditions, collected the lysate, and measured firefly luciferase activity (TOPFLASH), as well as Renilla luciferase activity (Renilla) as endogenous control.

### 2.8. Statistical Analysis

Data were expressed as means ± standard deviations. Statistical analysis was performed using JMP Pro 14 software (SAS Institute, Cary, NC, USA). Statistical differences were analyzed with the Student’s *t*-test for continuous variables and the chi-squared test for noncontinuous data. Survival curves were generated using the Kaplan–Meier method and assessed using the log-rank test. A value of *p* < 0.05 was considered statistically significant.

## 3. Results

### 3.1. Immunohistochemistry for LRP6 and β-Catenin in CRC

**Figure 1:**
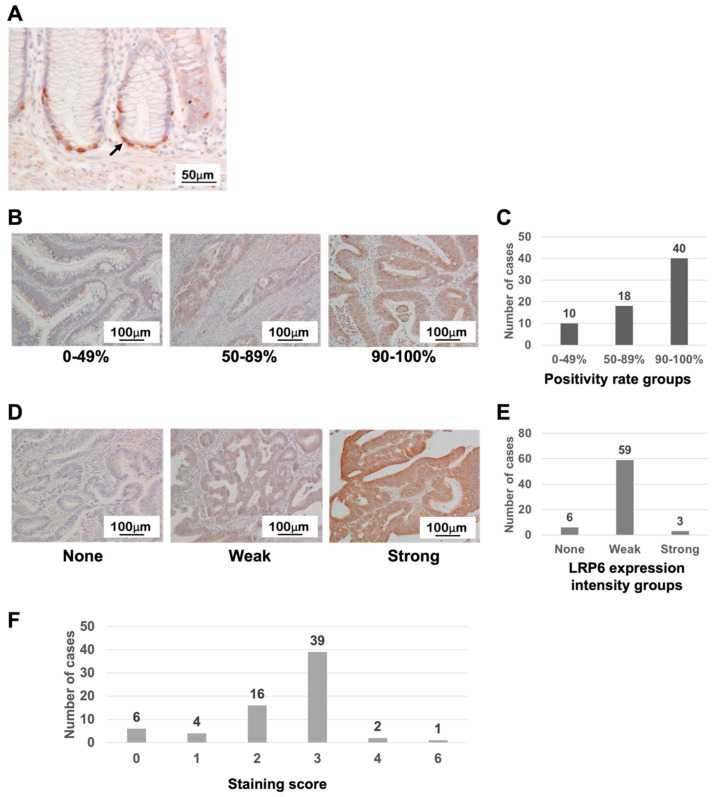
LRP6 expression in normal colon and CRC tissues. (**A**) LRP6 expression in normal colon mucosa. (**B**,**C**) Classification of the positivity of LRP6 expression in CRC. (**D**,**E**) Classification of LRP6 expression intensity in CRC. (**F**) Distribution of the staining score, calculated by multiplying the intensity and positivity scores.

In normal mucosa of colon tissues, LRP6-positive cells were generally localized at the bottom of the colonic epithelium (Figure 1A, arrow), whereas CRC tissue samples expressed the LRP6 protein mainly in the cytoplasm to varying extents (Figure 1B,C). With regard to staining intensity, most CRC samples showed weak staining for LRP6 (59/68: 86.8%) (Figure 1D,E). Most CRC samples also had a staining score of 2 or 3 (55/68: 80.9%) (Figure 1F).

**Figure 2:**
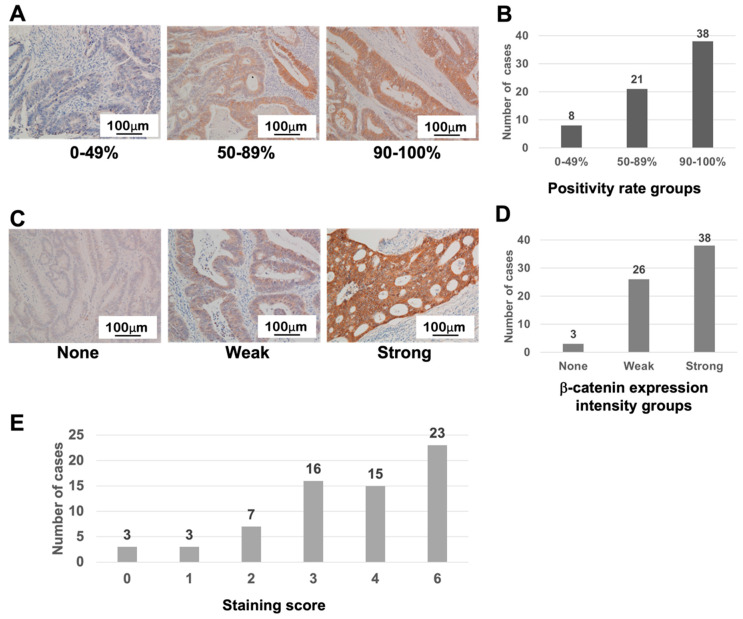
β-catenin expression in CRC tissues. (**A**,**B**) Classification of the positivity of β-catenin expression in CRC. (**C**,**D**) Classification of β-catenin expression intensity in CRC. (**E**) Distribution of the staining score, calculated by multiplying the intensity and positivity scores.

In contrast to the results for LRP6 staining, many cases showed strong β-catenin expression (strong: 2) at a high incidence (90–100%), mainly in the cytoplasm (Figure 2A–D). As a result, staining scores were relatively higher than for LRP6, and 38 of 67 cases (56.7%) had scores of 4 or 6 points (Figure 2E).

### 3.2. Immunohistochemistry for LRP6 and β-Catenin in ESCC

**Figure 3:**
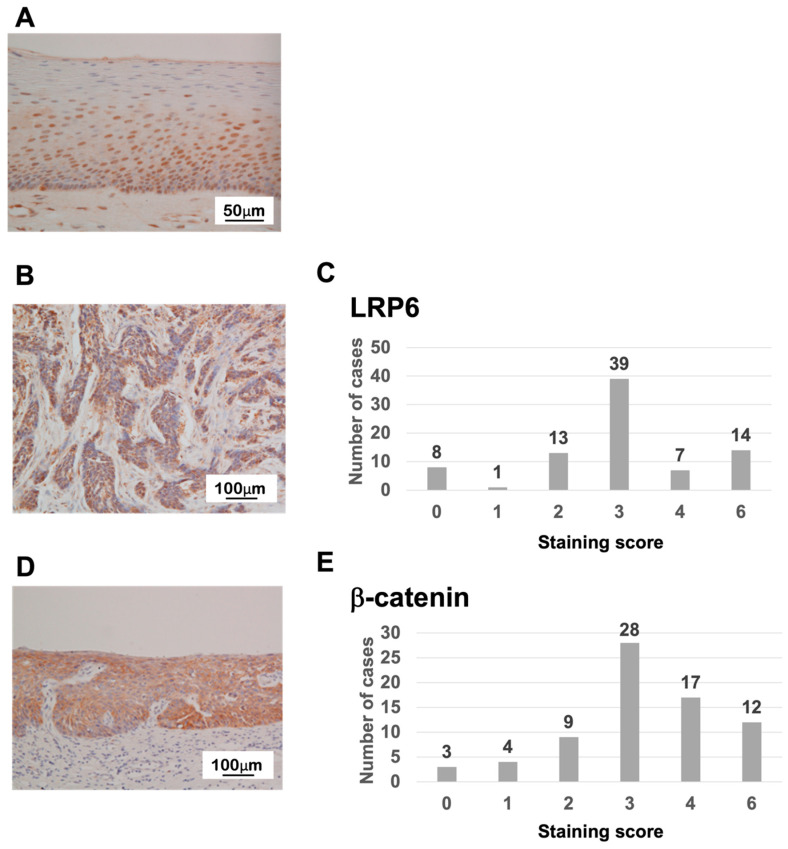
LRP6 and β-catenin expression in normal esophageal and ESCC tissues. (**A**) LRP6 expression in normal esophageal tissues. (**B**,**C**) Distribution of the LRP6 staining score, calculated by multiplying the intensity and positivity scores. (**D**,**E**) Distribution of the β-catenin staining score, calculated by multiplying the intensity and positivity scores.

In normal squamous epithelium of esophageal tissues, LRP6 expression was found mainly in the parabasal layer (Figure 3A). In ESCC tissue samples, expression of the LRP6 protein was similar to that for CRC, and 21 of 82 cases (25.6%) were classified with a high staining score of 4 or 6 (Figure 3B,C and Supplementary Figure S3). Many samples showed overall cytoplasmic β-catenin staining similar to CRC, but a greater proportion of ESCC vs. CRC cases had weak intensity. We found that 29 out of 73 cases (39.7%) had a staining score of 4 or 6 for β-catenin in ESCC (Figure 3D,E and Supplementary Figure S3).

### 3.3. Influence of LRP6 on Wnt/β-Catenin Signaling Activity

**Figure 4:**
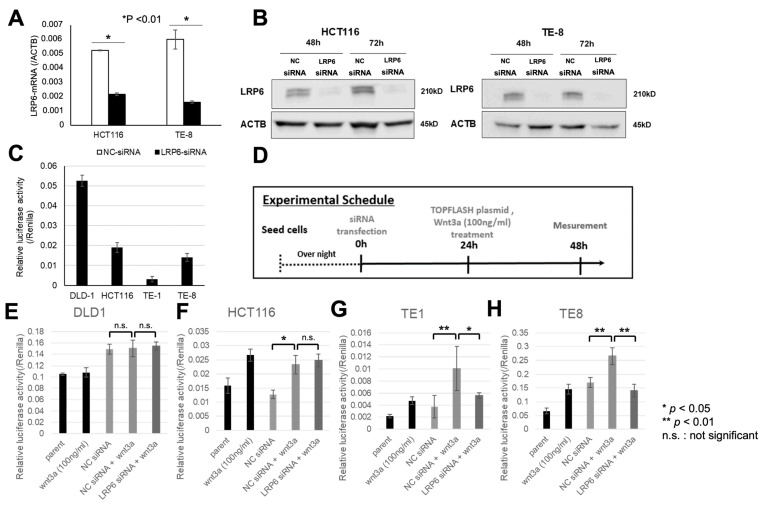
Effects of LRP6 on Wnt/β-catenin signaling activity. (**A**) Expression of LRP6 mRNA in CRC and ESCC cell lines, as measured by qRT-PCR. We used ACTB as endogenous control. * *p* < 0.01. (**B**) The protein level of LRP6 in CRC and ESCC cell lines. We used ACTB as endogenous control. (**C**) Wnt/β-catenin signaling activity under unstimulated conditions in CRC and ESCC cell lines. (**D**) Experimental plan for investigating signaling activity by adding LRP6-siRNA or Wnt3a to the CRC and ESCC cell lines. (**E**–**H**) Wnt/β-catenin signaling activity using CRC and ESCC cell lines. We used Renilla as endogenous control. * *p* < 0.05, ** *p* < 0.01; n.s.: not significant.

With the introduction of LRP6-siRNA into HCT116 and TE-8, LRP6 mRNA expression decreased compared with expression in the control group receiving NC-siRNA (Figure 4A). Likewise, protein levels decreased at 48 and 72 h (Figure 4B). Next, we investigated the relation between LRP6 expression and Wnt/β-catenin signaling activity in CRC and ESCC cell lines. Under the unstimulated condition, Wnt/β-catenin signaling activity in TE-1 and TE-8 cells was lower than in CRC cells (Figure 4C).

As shown in Figure 4D, we performed TOPFLASH assay. In DLD-1 cells, which carry the homozygous mutation in *APC*, we found no response to stimulation with Wnt3a, along with unchanged Wnt/β-catenin signaling under LRP6 suppression (Figure 4E). In contrast, in HCT116 cells, which carry the heterozygous mutation in *CTNNB1* encoding β-catenin, Wnt/β-catenin signaling activity significantly increased with Wnt3a stimulation and did not decline with LRP6 suppression through the introduction of LRP6-siRNA (Figure 4F).

In TE-1 and TE-8 cells, which do not carry a mutation in *APC* or *CTNNB1*, signaling activity increased under Wnt3a stimulation in both cell lines (*p* < 0.01), and introduction of LRP6-siRNA significantly dampened this increase (*p* < 0.01 or 0.05) (Figure 4G,H).

### 3.4. Relationship between the Expression of LRP6 or β-Catenin Protein and Prognosis in CRC or ESCC

To explore the relationship of LRP6 expression and β-catenin protein with clinicopathological correlation and prognosis in CRC and ESCC, we divided the cases with clinical follow-up data by high LRP6 expression (staining score >3) and low LRP6 expression (staining score < 2). Similarly, we performed analyses with two other groups, one with high β-catenin expression (staining score > 4) and one with low β-catenin expression (staining score < 3). The clinicopathological status of 58 CRC patients and 68 ESCC patients, which was stratified by LRP6 and β-catenin expression level, are shown in Table 1 and Table 2. High expression of LRP6 in CRC was significantly associated with lymphatic invasion (*p* = 0.035, Table 1). Kaplan–Meier survival curves indicated no significant difference between either set of compared groups in relapse-free survival (RFS) or overall survival (OS) (Supplementary Figures S4 and S5).

## 4. Discussion

### 4.1. The Function of LRP6 May Differ Depending on Genetic Abnormalities in the Wnt/β-Catenin Pathway

In CRC, gene mutations in proteins downstream of the Wnt/β-catenin signaling pathway, such as *APC* (67%), *CTNNB1* (6%), and *AXIN2* (5%), are often observed [24]. Liver cancer predominantly has mutations in *CTNNB1* (25%) and *AXIN1* (8%) genes [24]. On the other hand, breast [25] and pancreatic cancer [26] cases rarely have mutations in these genes. Although overexpression of LRP6 is observed in these types of cancer [27], it is not known whether the function of LRP6 differs depending on genetic abnormalities in the Wnt/β-catenin pathway.

We performed an LRP6 knockdown in CRC and ESCC cells to investigate how LRP6 expression affects Wnt/β-catenin signaling. In the luciferase reporter assay using the TOPFLASH plasmid to evaluate Wnt/β-catenin signaling, we found almost no change under Wnt3a stimulation or LRP6 knockdown in DLD-1 cells carrying a homozygous *APC* mutation. One possible explanation is that the signal was activated in DLD-1 because of an *APC* mutation affecting the downstream signaling in the Wnt/β-catenin pathway, so that the upstream factor LRP6 did not significantly affect this activity. To confirm whether LRP6 knockdown only affects the Wnt/β-catenin pathway when it is not constitutively active, an experiment with overexpression of APC protein in DLD-1 to reduce Wnt/-catenin pathway activation may be helpful. Because APC is a huge protein, the center third of APC (cAPC), which contains both β-catenin and AXIN binding domain and is sufficient to promote degradation of β-catenin protein [28], may be useful for this experiment. HCT116 cells, which carry a heterozygous mutation (one wild-type allele and one mutant allele with inactivation of SER45) in *CTNNB1*, responded to stimulation by Wnt3a but not to LRP6 knockdown. The wild-type allele in HCT116 provided susceptibility to Wnt3a, but the mutant SER45 allele likely caused accumulation of β-catenin. These results suggest that LRP6 is not important for the activation of Wnt/β-catenin signaling in CRC, which harbors genetic abnormalities in this pathway. There are several controversial studies showing whether LRP6 expression contributes to Wnt/β-catenin signal activation in CRC with *APC* and *CTNNB1* mutations. Raisch et al. demonstrated that LRP6 did not affect tumorigenesis in *APC^Min/+^* mice, and they also showed that LRP6 knockdown did not affect the colony formation activity and cell growth in CRC cells with *APC* or *CTNNB1* mutations, suggesting that LRP6 is dispensable for tumorigenesis induced by an aberrant Wnt/β-catenin pathway [29]. Chen et al. showed that LRP6 knockout in CRC cells with *APC* mutations did not alter the activity of the Wnt/β-catenin pathway [30]. On the other hand, Yao et al. reported that LRP6 overexpression in CRC cells activated the Wnt/β-catenin pathway [19]. Saito-Diaz et al. showed that LRP6 is required for activation of the Wnt/β-catenin pathway in CRC cells with *APC* mutations, but not in CRC cells with *CTNNB1* mutations [31], and Cabel et al. confirmed this result with a single cell analysis [32]. Our results support the former studies.

Unlike DLD-1 and HCT116 cells, the TE-1 and TE-8 ESCC cell lines without mutations in *APC* and *CTNNB1* show no enhanced signal activity downstream of the Wnt/β-catenin pathway. Therefore, they responded to Wnt3a stimulation and showed a decrease in this response under LRP6-siRNA exposure, suggesting that LRP6 is important for regulating the Wnt/β-catenin pathway in ESCC. Our results support previous findings that LRP6 is associated with cell migration, invasion, and epithelial-to-mesenchymal transition in ESCC cell lines [33].

Taken together, our results suggest that the function of LRP6 may differ depending on genetic abnormalities in the Wnt/β-catenin pathway (Supplementary Figure S6).

### 4.2. The Expression of LRP6 and β-Catenin in CRC and ESCC

LRP6 is a membrane protein associated with the Wnt/β-catenin signaling pathway (Supplementary Figure S1) and an important mediator of the intestinal stem cell niche [29]. It contributes to maintenance of the intestinal crypt structure and transmits signals downstream by binding to Wnt ligands [34]. Our immunostaining showed that in normal colonic epithelial tissue, LRP6-positive cells were observed at the crypts of the glandular duct that are sites of active cell division, with increased Wnt/β-catenin signaling. In normal esophageal tissue, LRP6-positive cells are mainly located in the parabasal layer; consistent with this layer is the squamous epithelium proliferative zone where stem cells are present [35].

In cancer, many CRC samples demonstrate a high positivity rate for β-catenin and strong staining intensity, which is consistent with previous reports. Although the staining intensity for LRP6 was not as strong in many CRC samples, the positivity rate was high. This result supports the idea that LRP6 may not be important for the activation of the Wnt/β-catenin pathway in CRC with genetic abnormalities such as *APC* or *CTNNB1* mutations.

In contrast, for β-catenin staining scores in ESCC samples that were lower than in CRC samples and related to LRP6, the positivity rate did not differ significantly compared with CRC samples, but the number of ESCC samples with strong intensity tended to increase. This result suggests that increased expression of LRP6 is associated with the activation of the Wnt/β-catenin pathway in ESCC.

These results are consistent with our in vitro data and further support the idea that LRP6 functions differentially in cancers depending on the presence or absence of genetic abnormalities in the Wnt/β-catenin pathway.

### 4.3. Clinical Significance of LRP6 Expression in CRC and ESCC

By analyzing the relevance of LRP6 expression to prognosis and clinicopathological parameters, we found a significant correlation between high LRP expression and lymphatic invasion in CRC, suggesting that LRP6 is associated with the invasive activity of CRCs. This is consistent with the studies that show LRP6 is associated with metastasis and poor prognosis in multiple types of cancers such as breast cancer [36], liver cancer [37], and oral squamous cell carcinoma [38]. In addition, Yao et al. reported that LRP6 promoted the migration of CRC cells through regulation of cytoskeleton dynamics [19]. However, as described above, this group showed that LRP6 overexpression activated the Wnt/β-catenin pathway even in CRC cells with *APC* or *CTNNB1* mutations. Therefore, further studies will be needed to reveal the underlying mechanism.

Although high LRP expression was significantly correlated with lymphatic invasion in CRC, Kaplan–Meier curves showed no significant difference in OS or RFS between groups with high vs. low LRP6 expression in CRC. This discrepancy may be because we analyzed only small number of samples for survival analysis in this study. Therefore, further investigation using a higher sample number will be required.

### 4.4. The Potential of LRP6 as a Therapeutic Target

Small molecule inhibitor, monoclonal antibody and modified peptides, which target the Wnt/β-catenin pathway have been developed and have proceeded to clinical trial [39]. The target of these drugs are various proteins in the Wnt/β-catenin pathway [40], such as ligands (e.g., WNT5a) [41], receptors and coreceptors (e.g., Fzd) [42], intracellular components (e.g., β-catenin) [43], and transcription cofactors (e.g., CBP) [44]. With regards to LRP6, Niclosamide [45], Salinomycin [46], and Rottlerin [47] are known to suppress LRP6 expression.

Our results suggest that LRP6 inhibition and other Wnt/β-catenin inhibitors may be effective for ESCC in case of LRP6 overexpression and upregulated Wnt/β-catenin pathway. Meanwhile, LRP6 inhibition may not be effective against CRC with genetic abnormalities in the Wnt/β-catenin pathway.

However, it is reported that LRP6 affects not only the Wnt/β-catenin pathway, but also other pathways such as the noncanonical Wnt pathway [48], Wnt-dependent stabilization of proteins (Wnt/STOP) pathway [49], G protein-coupled receptor (GPCR) pathway [50], and Hippo pathway [51]. These pathways are related to tumorigenesis and malignant phenotype of CRC. For example, overexpression of Wnt11, which activates the noncanonical Wnt pathway promotes the proliferation, invasion, and migration of CRC cell lines [52]. Some GPCRs such as S1PR3, S1PR5, and AT1R are associated with tumorigenesis, proliferation, invasion, and migration of CRC cells [53]. Cho et al. reported that upregulation of Hippo pathway genes is related to poor prognosis in patients with CRC [54]. Therefore, LRP6 may be involved in the malignant phenotype of CRC cells via these pathways.

If LRP6 overexpression is associated with the malignant phenotype of CRC independent of the Wnt/β-catenin pathway, it is possible that the combined inhibition of LRP6 with an intracellular component or transcription cofactors of the Wnt/β-catenin pathway may have additive or synergistic effects. Further studies will be needed to investigate this possibility.

Nucleic acid medicine, including small interference RNA (siRNA) and microRNA (miRNA), is considered a next-generation cancer therapy [55]. We previously showed that microRNA-487b (miR-487b) decreased LRP6 expression by directly binding to the 3′-untranslational region (UTR) of LRP6 mRNA, and miR-487b treatment suppressed the proliferation and invasion of CRC cells with *APC* or *CTNNB1* mutations [56]. We developed an improved in vivo drug delivery system, which is named as inorganic nanoparticle device (iNaD), and we showed the effective antitumor effect of miRNA encapsuled into iNaD [57]. Therefore, the combinational treatment of iNaD-miR-487b and Wnt inhibitors (e.g., β-catenin inhibitor, CBP inhibitor) may be a new therapeutic strategy for CRC with genetic abnormalities in the Wnt/β-catenin pathway.

## 5. Conclusions

Our findings indicate that LRP6 could behave differently in different cancers based on genetic abnormalities related to the Wnt/β-catenin pathway. Further studies are needed to elucidate the function of LRP6 in cancer and characterize its potential as a therapeutic target.

## Figures and Tables

**Table 1:** Comparison of clinicopathological status stratified by LRP6 or β-catenin expression in CRC tissue samples.

**Characteristic**		**LRP6 High (n = 29)**	**LRP6 Low (n = 29)**	***p* Value**
Age	Average ± SD	64.6 ± 11.72	66.1 ± 9.52	0.591
Sex	Male	17	20	0.412
	Female	12	9	
Lymphatic invasion	Positive	17	9	0.035
	Negative	12	20	
Venous invasion	Positive	6	2	0.253
	Negative	23	27	
Lymph node metastasis	Positive	6	3	0.253
	Negative	23	26	
Distant metastasis	Positive	3	1	0.612
	Negative	26	28	
Degree of differentiation *	Well/Mod	28	28	1.000
	Poor/Muc	1	1	
Tumor location	Colon	14	12	0.598
	Rectum	15	17	
Depth of invasion **	~mp	23	22	0.753
	ss~	6	7	
**Characteristic**		**β-Catenin High (n = 24)**	**β-Catenin Low (n = 34)**	***p* Value**
Age	Average ± SD	63.2 ± 9.16	66.9 ± 11.41	0.192
Sex	Male	14	23	0.467
	Female	10	11	
Lymphatic invasion	Positive	12	14	0.506
	Negative	12	20	
Venous invasion	Positive	5	3	0.255
	Negative	19	31	
Lymph node metastasis	Positive	4	5	1.000
	Negative	20	29	
Distant metastasis	Positive	3	1	0.297
	Negative	21	33	
Degree of differentiation *	Well/Mod	23	33	1.000
	Poor/Muc	1	1	
Tumor location	Colon	10	16	0.684
	Rectum	14	18	
Depth of invasion **	~mp	18	27	0.692
	ss~	6	7	

* Well: well differentiated adenocarcinoma, Mod: moderately differentiated adenocarcinoma, Poor: poorly differentiated adenocarcinoma, Muc: mucinous carcinoma. ** mp: muscularis propria, ss: subserosa.

**Table 2:** Comparison of clinicopathological status stratified by LRP6 or β-catenin expression in ESCC tissue samples.

**Characteristic**		**LRP6 High (n = 49)**	**LRP6 Low (n = 19)**	***p* Value**
Age	Average ± SD	70.0 ± 8.54	69.6 ± 8.73	0.8675
Sex	Male	43	13	0.0606
	Female	6	6	
T stage	T1, T2	30	15	0.2537
	T3, T4	19	4	
Lymphatic invasion	Positive	34	9	0.1803
	Negative	14	8	
Venous invasion	Positive	9	5	0.2949
	Negative	39	11	
Lymph node metastasis	Positive	30	9	0.300
	Negative	19	10	
Distant metastasis	Positive	2	0	1.000
	Negative	47	18	
Degree of differentiation *	Well/Mod	38	17	0.3251
	Poor	11	2	
Tumor size (max length)	mm, Average ± SD	47.1 ± 25.86	40.5 ± 25.98	0.4187
**Characteristic**		**β-Catenin High (n = 26)**	**β-Catenin Low (n = 42)**	***p* Value**
Age	Average ± SD	70.7 ± 7.95	69.4 ± 8.92	0.537
Sex	Male	20	36	0.355
	Female	6	6	
T stage	T1, T2	16	29	0.524
	T3, T4	10	13	
Lymphatic invasion	Positive	18	25	0.249
	Negative	6	16	
Venous invasion	Positive	7	7	0.215
	Negative	16	34	
Lymph node metastasis	Positive	16	23	0.583
	Negative	10	19	
Distant metastasis	Positive	1	1	1.000
	Negative	24	41	
Degree of differentiation *	Well/Mod	19	36	0.198
	Poor	7	6	
Tumor size (max length)	mm, Average ± SD	51.5 ± 16.52	41.9 ± 29.43	0.133

* Well: well differentiated adenocarcinoma, Mod: moderately differentiated adenocarcinoma, Poor: Poorly differentiated adenocarcinoma.

## Data Availability

Not applicable.

## Supplementary Materials

The following supporting information can be downloaded at: https://www.mdpi.com/article/10.3390/life13030615/s1, Figure S1: Schematic illustration of Wnt/b-catenin pathway and its association with LRP6. Figure S2: Positive and Negative control of LRP6 immunostaining. (A) Positive control: LRP6 is expressed in a breast cancer. (B) Negative control: Phosphate-buffered saline was used instead of the primary antibody. Figure S3: LRP6 and β-catenin expression in ESCC tissues. (A, B) Classification of the positivity of LRP6 expression in ESCC. (C, D) Classification of LRP6 expression intensity in ESCC. (E, F) Classification of the positivity of β-catenin expression in ESCC. (G, H) Classification of β-catenin expression intensity in ESCC. Figure S4: Survival analysis in CRC tissues. (A) Overall survival of CRC patients with LRP6 high or low expression. (B) Relapse-free survival of CRC patients with LRP6 high or low expression. (C) Overall survival of CRC patients with β-catenin high or low expression. (D) Relapse-free survival of CRC patients with β-catenin high or low expression. Figure S5: Survival analysis in ESCC tissues. (A) Overall survival of ESCC patients with LRP6 high or low expression. (B) Relapse-free survival of ESCC patients with LRP6 high or low expression. (C) Overall survival of ESCC patients with β-catenin high or low expression. (D) Relapse-free survival of ESCC patients with β-catenin high or low expression. Figure S6: Schematic illustration of the relationship between Wnt/b-catenin pathway and LRP6 in CRC and ESCC.
