# Supplementary material for: The Relationship between LRP6 and Wnt/β-Catenin Pathway in Colorectal and Esophageal Cancer
Source: Life (Basel). 2023 Feb 23;13(3):615. doi: 10.3390/life13030615 (PMC10057833; doi:10.3390/life13030615)
Supplement: Supplementary file 1 [file life-13-00615-s001.zip › life-2197124-supplementary.pdf]

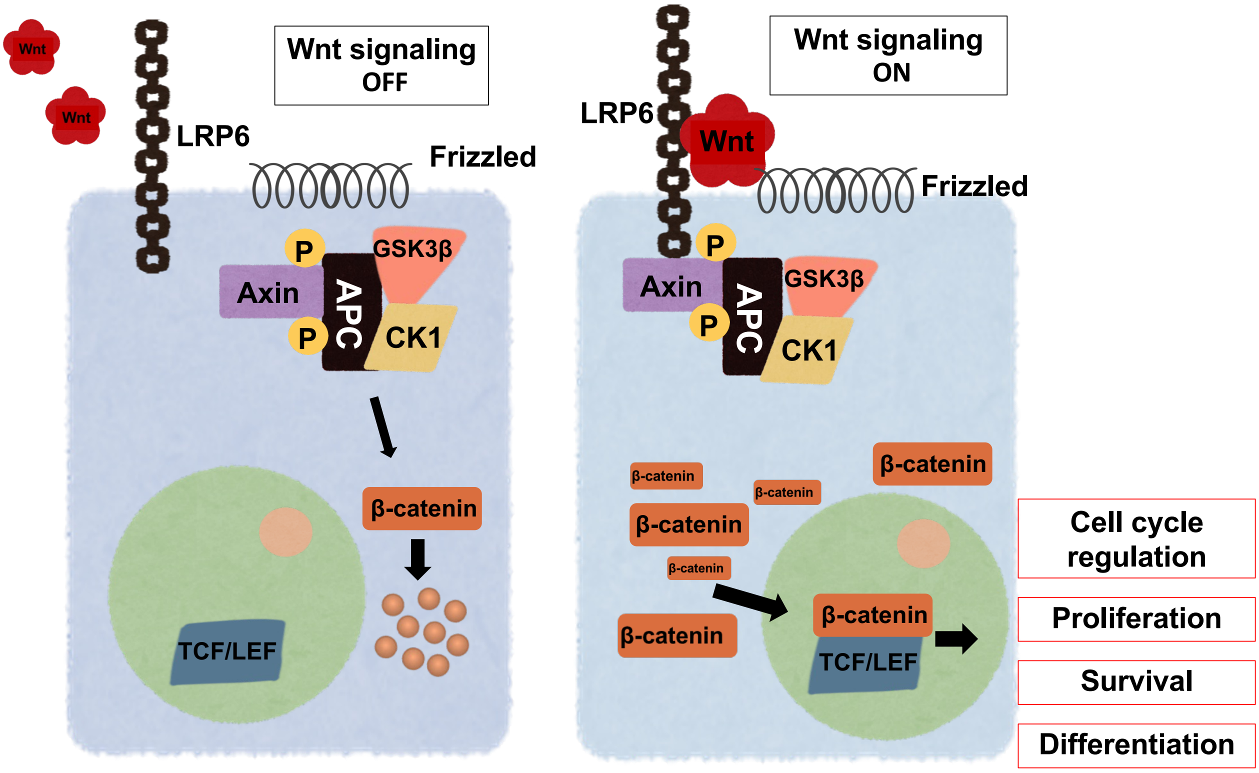

Supplementary Fig. S1
Schematic illustration of Wnt/β-catenin pathway and its association with LRP6.

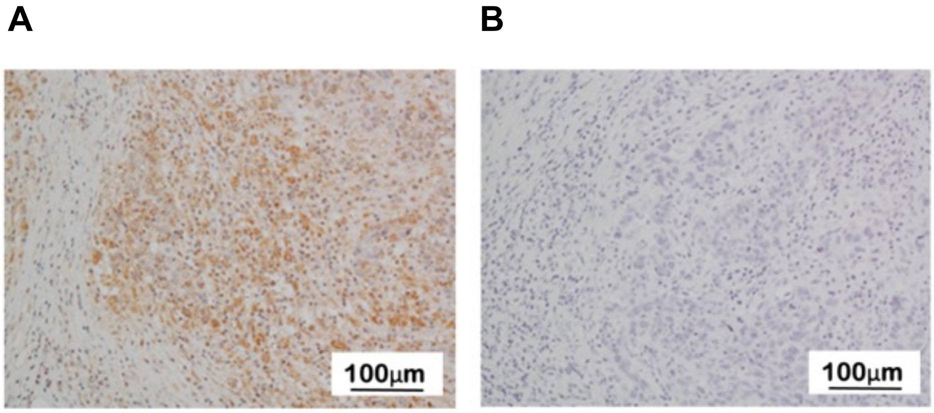

Supplementary Fig. S2

Positive and Negative control of LRP6 immunostaining.

(A) Positive control: LRP6 is expressed in a breast cancer.

(B) Negative control: Phosphate-buffered saline was used instead of the primary antibody.

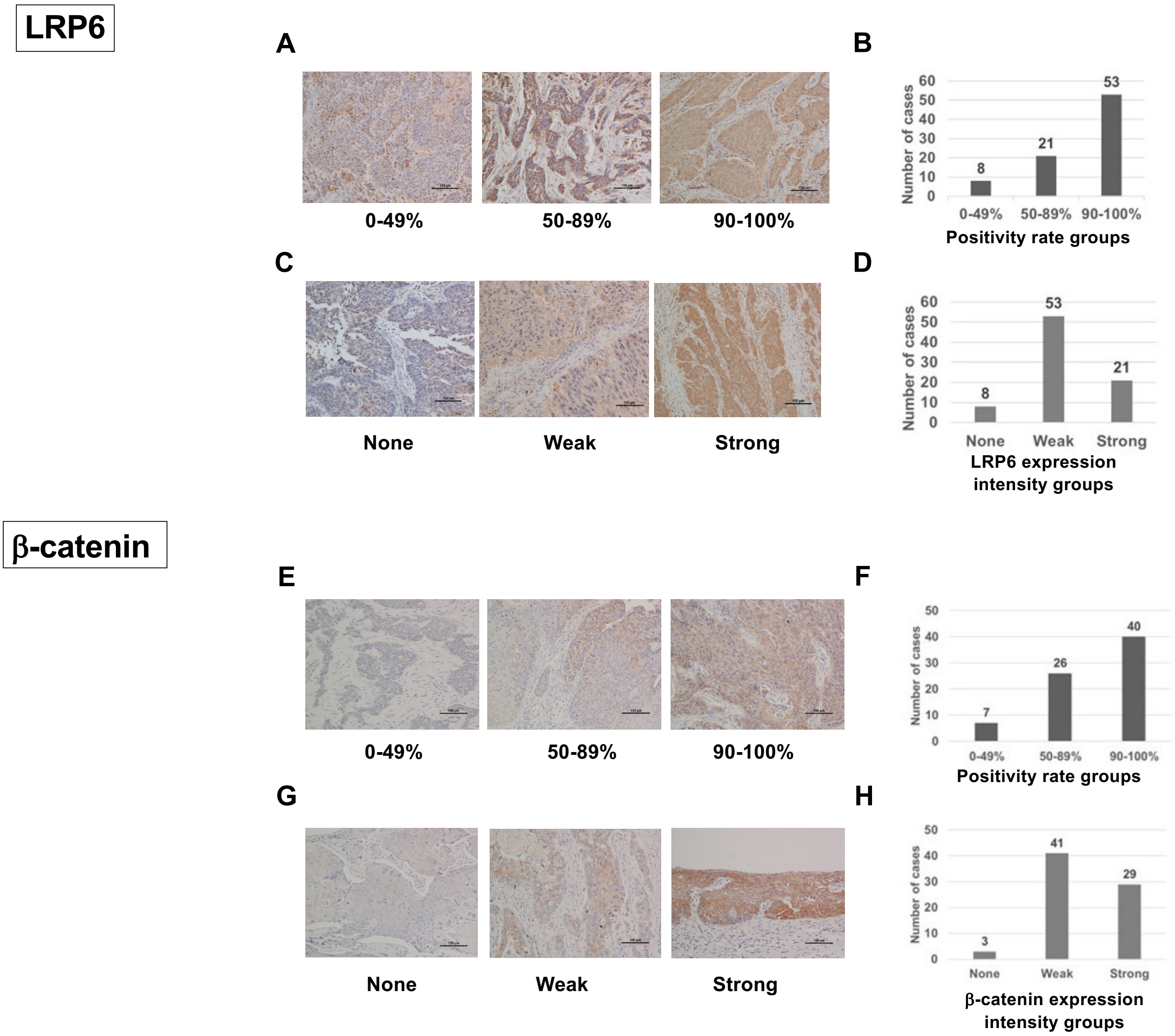

Supplementary Fig. S3

LRP6 and β-catenin expression in ESCC tissues.

(A, B) Classification of the positivity of LRP6 expression in ESCC. (C, D) Classification of LRP6 expression intensity in ESCC. (E, F) Classification of the positivity of β-catenin expression in ESCC. (G, H) Classification of β-catenin expression intensity in ESCC.

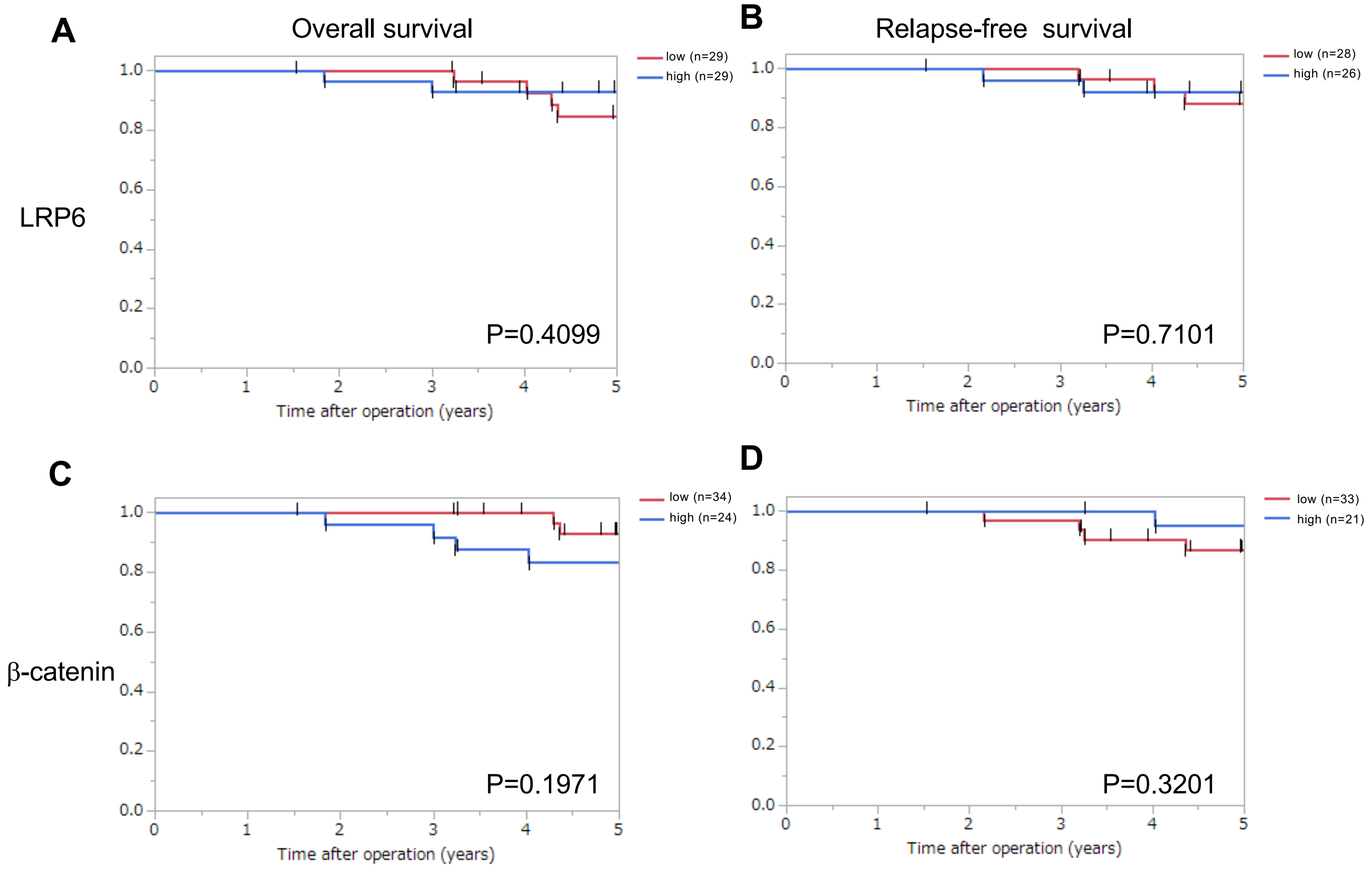

Supplementary Fig. S4

Survival analysis in CRC tissues.

(A) Overall survival of CRC patients with LRP6 high or low expression. (B) Relapse-free survival of CRC patients with LRP6 high or low expression. (C) Overall survival of CRC patients with β-catenin high or low expression. (D) Relapse-free survival of CRC patients with β-catenin high or low expression.

Supplementary Fig. S4

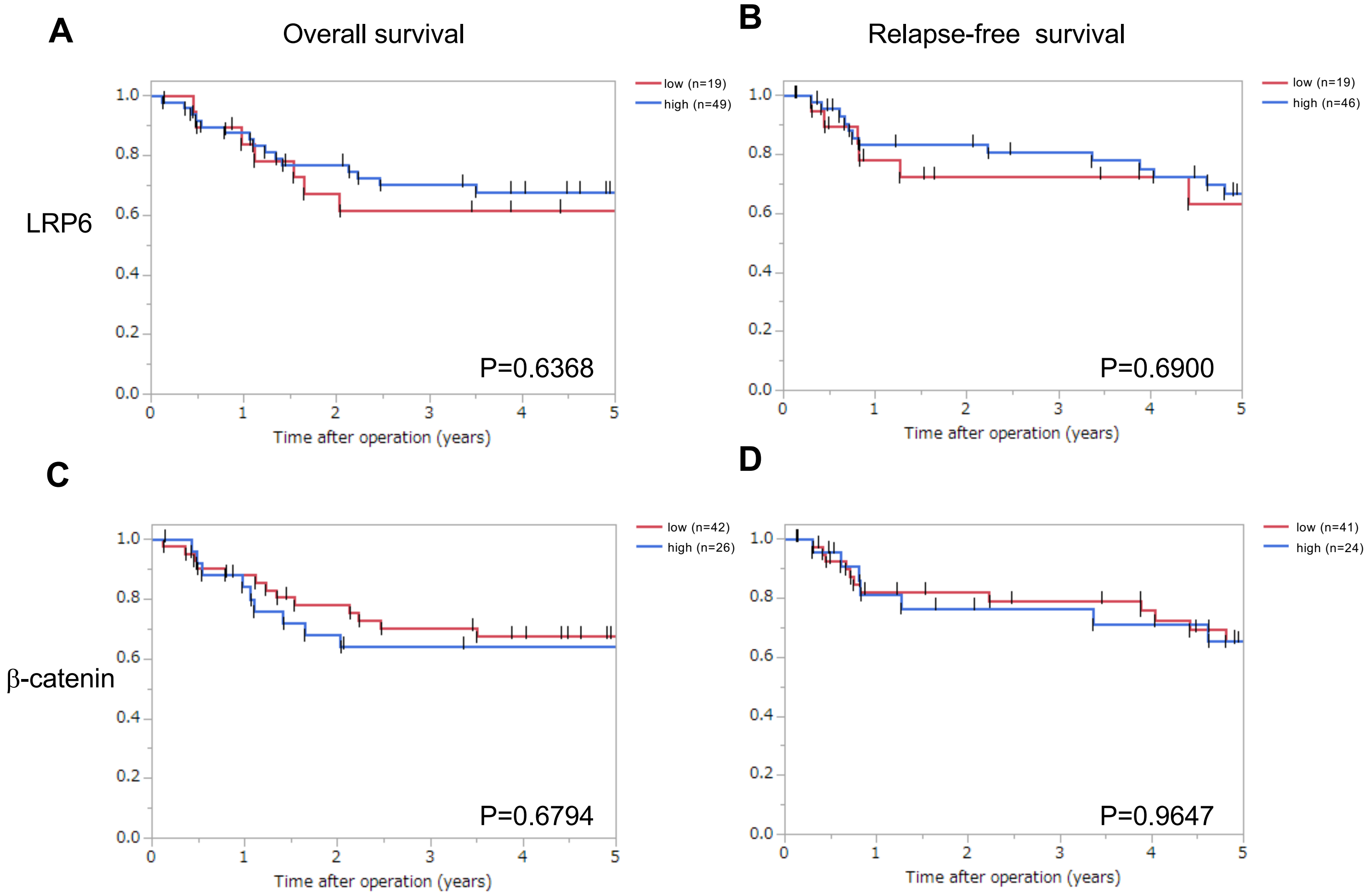

Supplementary Fig. S5

Survival analysis in ESCC tissues.

(A) Overall survival of ESCC patients with LRP6 high or low expression. (B) Relapse-free survival of ESCC patients with LRP6 high or low expression. (C) Overall survival of ESCC patients with β-catenin high or low expression. (D) Relapse-free survival of ESCC patients with β-catenin high or low expression.

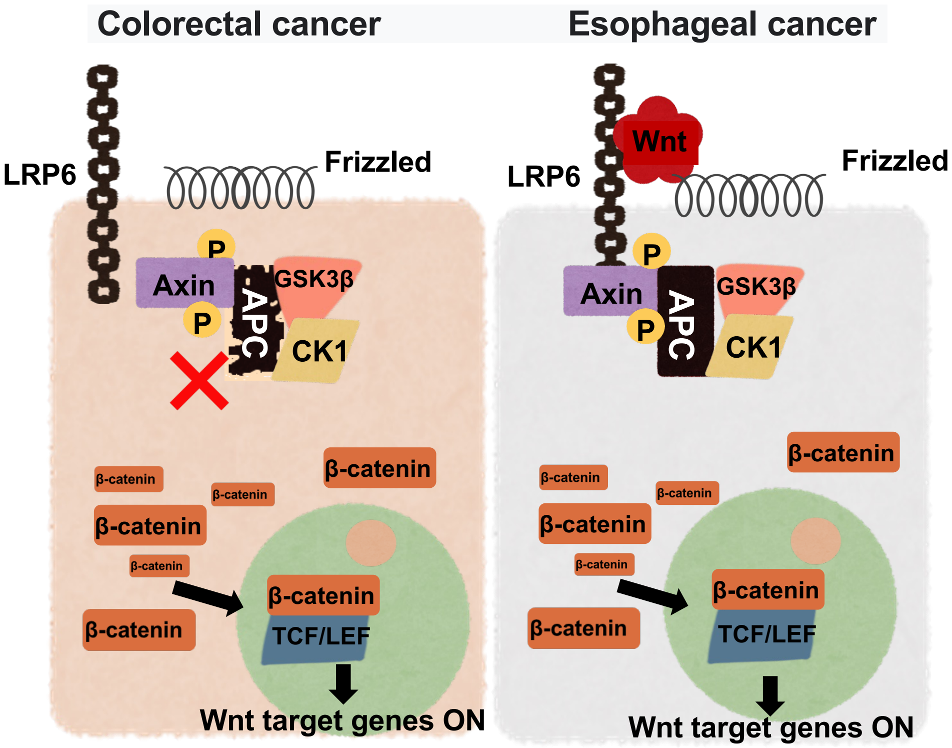

Supplementary Fig. S6
Schematic illustration of the relationship between Wnt/β-catenin pathway and LRP6 in CRC and ESCC.
